# Reversible Multi-Mode Optical Modification in Inverse-Opal-Structured WO_3_: Yb^3+^, Er^3+^ Photonic Crystal

**DOI:** 10.3390/ma17102436

**Published:** 2024-05-18

**Authors:** Bokun Zhu, Keliang Ruan, Cherkasova Tatiana, Yangke Cun

**Affiliations:** 1 College of Materials Science and Engineering, Kunming University of Science and Technology, Kunming 650093, China; zbk1101@163.com (B.Z.); 18705811258@163.com (K.R.); 2 School of Chemistry and Oil and Gas Technology, Kuzbas National Technical University, 650026 Kemerovo, Russia; ctg.htnv@kuzstu.ru

**Keywords:** upconversion, inverse opal structure, reversible modulation

## Abstract

Reversible optical regulation has potential applications in optical anti-counterfeiting, storage, and catalysis. Compared to common power materials, the reverse opal structure has a larger specific surface area and an increased contact area for optical regulation, which is expected to achieve higher regulation rates. However, it is difficult to achieve reversible and repeatable regulation of the luminescent properties of photonic crystals, especially with the current research on the structural collapse of photonic crystals. In this work, WO_3_: Yb^3+^, Er^3+^ inverse photonic crystals were prepared by the template approach, and reversible multi-mode optical modification was investigated. Upon heat treatment in a reducing atmosphere or air, the color of the photonic crystals can reversibly change from light yellow to dark green, accompanied by changes in absorption and upconversion of luminescence intensity. The stability and fatigue resistance of this reversible optical modification ability were explored through cyclic experiments, providing potential practical applications for photocatalysis, optical information storage, and electrochromism.

## 1. Introduction

Lanthanide-ion-doped inorganic luminescent materials are extensively used in photoelectric fields, including in photocatalysis, optical communication, and optical storage [1] due to the abundant luminescent energy levels of lanthanide ions, which contribute to achieving excellent luminescent performance. Rapid advancement in science and technology has led to an exponential increase in the demand for innovative luminescent materials in the aforementioned application domains [2,3,4,5]. The modification of optical performance is a pioneering strategy with significant practical implications and theoretical value. Recently, several common optical performance modulation techniques have emerged, for instance, co-doping a matrix or modifying the concentration of specific ions to disrupt the local symmetry of the crystal field to improve rare earth luminescence [6] and adjusting the doping concentration of rare earth ions to adjust the energy transfer of rare earth ions [7,8]. Additionally, using more complex photonic structures like constructing core–shell structures to govern energy transfer [9,10], using photonic crystals with photonic band gaps for selective luminescence enhancement [11], or using the coupling of surface plasmons and photonic crystal effects to enhance luminescence [12,13].

In the aforementioned techniques, photonic crystal materials have demonstrated distinctive advantages as optical materials because of their well-ordered, three-dimensional, macroscopic, porous structure with interconnected pores, wherein each larger air void corresponds to three small dark regions in the lower layer [14,15]. The structure of photonic crystals is characterized by a periodic arrangement, which gives rise to the presence of photonic bandgaps within this periodicity. By manipulating the lattice parameters and refractive indices of materials, precise control over the optical properties of photonic crystals can be achieved [16,17]. Leveraging the unique structure of photonic crystals to enhance the interaction between light and materials thereby augments the luminescent properties of materials, offering a compelling approach for modulating the optical characteristics of materials, and further expands their applications in fields such as optical storage and photocatalysis. Cheng Zhu et al. investigated the effect of the photonic bandgap on the luminescence characteristics of NaYF_4_:Yb, Tm@SiO_2_ photonic crystals [18]. However, it is difficult to achieve reversible and repeatable regulation of the luminescent properties of photonic crystals. Current studies still report the existence of a potential risk of structural collapse during optical modulation, which will significantly impact the reversibility and repeatability in practical applications [19]. Therefore, the development of lanthanide-ion-doped photonic crystals with reversible optical performance poses a crucial challenge.

In the field of photocatalysis, commonly employed metal oxide materials include TiO_2_, WO_3_, ZnO, SnO_2_, and niobium oxide nanowires, among other notable examples [20,21,22,23,24]. Through the absorption of light in the near-infrared wavelength range, these materials exhibit upconverted luminescent properties, thereby giving them potential applications in fields such as optical communication, optical storage, biomedicine, and photocatalysis. This study presents the design and fabrication techniques for opal gemstone-structured photonic crystal materials. The optical properties of the prepared WO_3_: Yb^3+^, Er^3+^ inverse photonic crystals are analyzed and explored through advanced optical testing and characterization techniques, providing crucial experimental evidence for comprehending the mechanism of their upconversion luminescence. Finally, this study explores the reversible modulation of absorption and upconversion luminescence performance, showcasing the technique’s potential applications in fields such as optical storage, optical sensors, biomarkers, and photocatalysis. These findings present novel concepts and prospects for photonic crystal materials.

## 2. Materials and Methods

### 2.1. Sample Preparation

(1) Preparation of Polystyrene (PS) Opal Templates:

A glass substrate was vertically immersed in a solution of monodisperse polystyrene microspheres with a concentration of 10% using the vertical precipitation method. Polystyrene microspheres then self-assembled on the substrate to form ordered opal templates.

(2) Preparation of Precursor Sol:

The precursor solutions WO_3_: x mol% Yb^3+^, 1 mol% Er^3+^ (x = 0.5, 1, 2, 3, 4) were prepared using (NH_4_)_6_H_2_W_12_O_40_·xH_2_O), Yb_2_O_3_, Er_2_O_3_, and HNO_3_ as raw materials. The stoichiometric Yb_2_O_3_ and Er_2_O_3_ were weighted to determine the different doping concentrations of Yb^3+^ and Er^3+^ ions and then dissolved in hot nitric acid to form Er(NO_3_)_3_ and Yb(NO_3_)_3_, which are then dissolved in anhydrous ethanol. (NH_4_)_6_H_2_W_12_O_40_·xH_2_O) was dissolved in deionized water and then added dropwise to an alcohol solution of lanthanide nitrates to obtain a precursor solution.

(3) Preparation of Inverse Opal Photonic Crystals:

The appropriate amount of precursor sol was slowly added to the PS opal template, and after complete filling, the PS microspheres were removed by sintering at 480 °C for 3 h to obtain WO_3_: Yb^3+^, Er^3+^ inverse opal photonic crystals.

**Figure 1:**
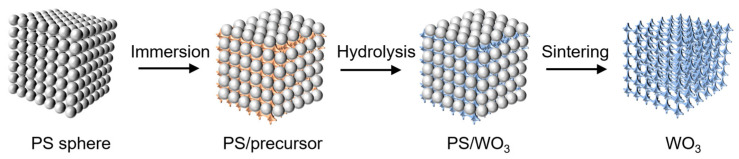
The preparation schematic diagram of WO_3_: Yb^3+^, Er^3+^ inverse opal photonic crystals.

The above sample preparation method is shown in Figure 1.

### 2.2. Characterization

The phase analysis of the samples was conducted using an X-ray diffractometer (XRD, D8 ADVANCE, Bruker, Mannheim, Germany). A field emission scanning electron microscope (FESEM, TESCAN MIRA LMS, Brno, Czech Republic) and energy dispersive spectroscopy (EDS) were employed to characterize the morphology and elemental distribution of the samples. X-ray photoelectron spectroscopy (XPS) was performed using a XPS spectrometer (Therma ESCALAB 250XI, Waltham, MA, USA) equipped with a vacuum radiation source emitting 300 W Al Kα. The absorption spectra of the samples were examined utilizing a spectrophotometer (HITACHI-U-4100, Tokyo, Japan). Upconversion luminescence measurements were performed employing a fluorescence spectrometer (HITACHI-F-7000, Japan).

## 3. Results and Discussion

### 3.1. The Phase and Structure of WO_3_: Yb^3+^, Er^3^ Inverse Photonic Crystals

**Figure 2:**
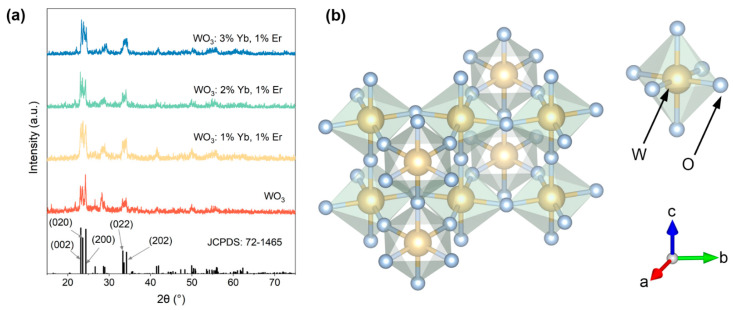
(**a**) XRD patterns of WO_3_ and WO_3_: x mol% Yb^3+^, 1 mol% Er^3+^ (x = 1, 2, 3); (**b**) schematic diagram of the crystal structure of WO_3_.

WO_3_ and WO_3_: Yb^3+^, Er^3+^ inverse photonic crystals with varying doping concentrations of Yb^3+^ were synthesized. The XRD patterns were performed on WO_3_ and WO_3_: x mol% Yb^3+^, 1 mol% Er^3+^ (x = 1, 2, 3), as illustrated in Figure 2a. All the XRD diffraction peaks exhibited excellent agreement with the standard PDF card JCPDS: 72-1465 [25] for monoclinic WO_3_, without any discernible impurity peaks. However, as the doping of lanthanide elements increases, the diffraction peak intensity of WO_3_ increases, which may be due to the doping of lanthanide elements changing the space group of WO_3_ [26]. Figure 2b shows a schematic diagram of the monoclinic phase WO_3_ crystal structure, consisting of an octahedral structure in which the tungsten atom occupies the center and is connected to six oxygen atoms.

**Figure 3:**
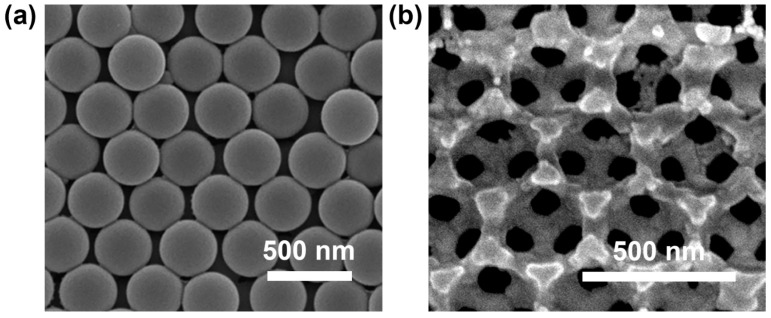
(**a**) The SEM image of the polystyrene microsphere opal template; (**b**) the SEM image of the WO_3_: 1 mol% Yb^3+^, 1 mol% Er^3+^ inverse photonic crystal.

Figure 3a illustrates the polystyrene microsphere opal template obtained through vertical deposition. The SEM image clearly revealed that the uniformly sized polystyrene microspheres formed a densely packed structure with a (111) planarly parallel face-centered cubic (FCC) crystal structure, ensuring a reliable foundation for subsequent photonic crystal synthesis. Furthermore, the surface morphology of the WO_3_: 1 mol% Yb^3+^, 1 mol% Er^3+^ inverse photonic crystals was analyzed using SEM images in Figure 3b, showing the successful synthesis of an inverted opal structure with a pore size of about 300 nm and a honeycomb-like, three-dimensional, macroporous, ordered structure.

**Figure 4:**
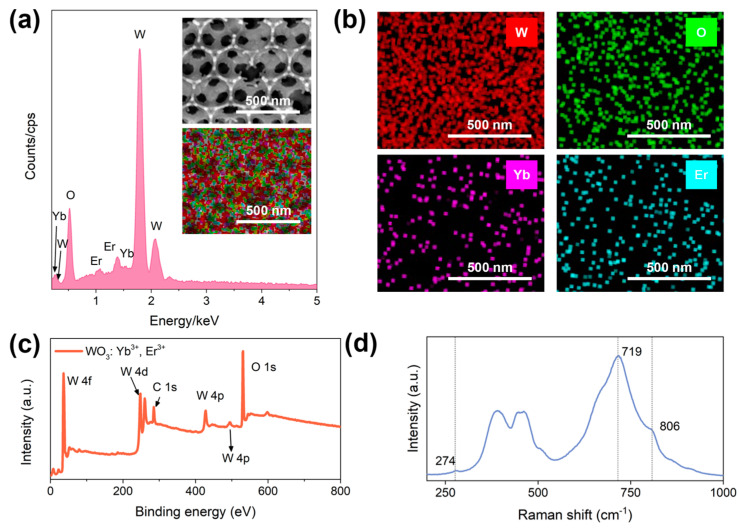
(**a**) The EDS spectrum of the WO_3_: 1 mol% Yb^3+^, 1 mol% Er^3+^ inverse photonic crystal and the corresponding (**b**) element mapping images; (**c**) the full XPS spectrum of the WO_3_: 1 mol% Yb^3+^, 1 mol% Er^3+^ inverse photonic crystal; (**d**) the Raman spectrum of the WO_3_: 1 mol% Yb^3+^, 1 mol% Er^3+^ inverse photonic crystal.

To further validate the successful doping of Yb^3+^ and Er^3+^ into the WO_3_ inverse photonic crystal, an EDS analysis was conducted on the WO_3_: Yb^3+^, Er^3+^ inverse photonic crystal. As shown in Figure 4a, the elements W, O, Yb, and Er were successfully detected in the sample at specific stoichiometric ratios, and elemental analysis showed a uniform distribution of W, O, Yb, and Er elements in WO_3_, indicating the successful doping of the lanthanide ions to Yb^3+^ and Er^3+^ into the WO_3_ inverse photonic crystal in Figure 4b. Figure 4c shows the complete XPS spectrum of the WO_3_: Yb^3+^, Er^3+^ inverse photonic crystal’s binding energies, which are further confirmed by comparison with the binding energies provided by the XPS standard spectrum handbook and database. Therefore, the binding energy provides convincing evidence for the successful synthesis of uniformly distributed WO_3_: Yb^3+^, Er^3+^ inverse photonic crystals. The Raman peaks of the WO_3_: 1 mol% Yb^3+^, 1 mol% Er^3+^ inverse photonic crystal located at 806 and 719 cm^−1^ are attributed to the stretching vibrations of δ(O-W-O), and the peak at 272 cm^−1^ belongs to the bending vibration of δ(O-W-O), exhibiting monoclinic structure characteristics [25].

### 3.2. The Optical Performance of WO_3_: Yb^3+^, Er^3+^ Inverse Photonic Crystal

**Figure 5:**
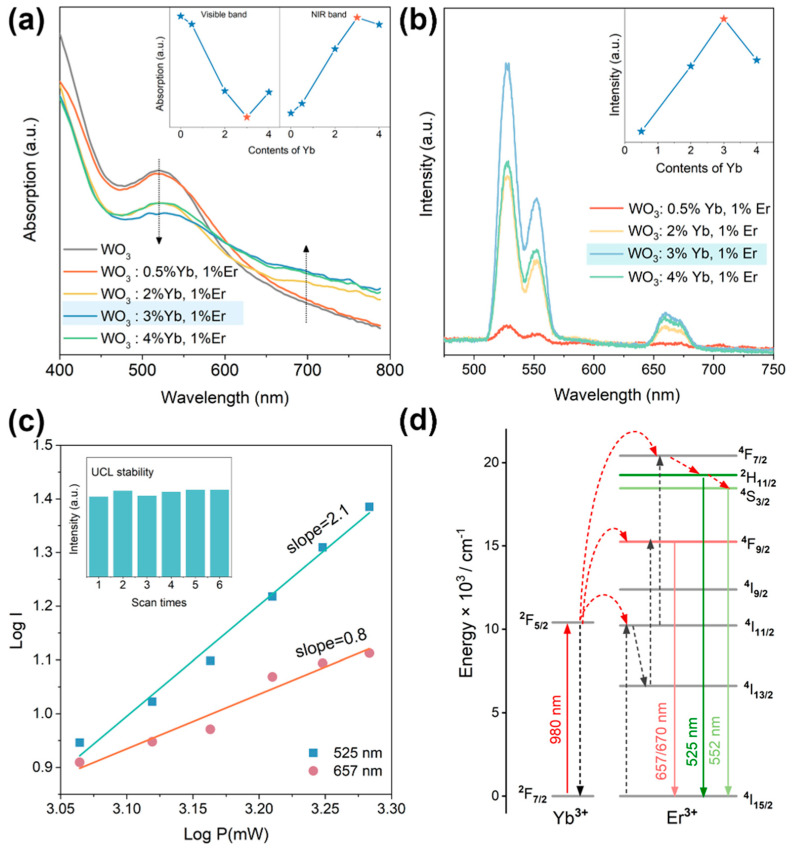
(**a**) Absorption spectra of WO_3_ and WO_3_: x mol% Yb^3+^, 1 mol% Er^3+^ (x = 0.5, 2, 3, 4); (**b**) the UCL spectra of WO_3_: x mol% Yb^3+^, 1 mol% Er^3+^ (x = 0.5, 2, 3, 4) excited at 980 nm; (**c**) the UCL stability (inset) and logarithmic plot of UCL intensity of WO_3_: 3 mol% Yb^3+^, 1 mol% Er^3+^; (**d**) diagram of the UCL mechanism of the WO_3_: Yb^3+^, Er^3+^ inverse photonic crystal.

Based on the successful synthesis confirmation of the photonic crystal WO_3_: Yb^3+^, Er^3+^, the absorption spectra were measured for both WO_3_ and WO_3_: x mol%Yb^3+^, 1 mol% Er^3+^ (x = 0.5, 2, 3, 4) inverse photonic crystals. As depicted in Figure 5a, the distinct absorption band observed in the range of 500–550 nm for WO_3_ belongs to the bandgap peak of WO_3_ inverse photonic crystals. Doping with lanthanides weakens the peak bandgap due to the decrease in the WO_3_ inverse photonic crystal’s structural order. However, with the increase in the Yb^3+^ concentration, the absorption ability of the sample in the near-infrared region above 700 nm gradually increase due to the increase in the number of oxygen vacancies in the matrix caused by the lanthanide doping [25]. DFT calculations indicate that the formation of oxygen vacancies in WO_3_ leads to the formation of localized energy levels in the forbidden band, with more W atoms transitioning to lower valence states. The transition of localized energy levels above the Fermi level facilitates a wide range of photoresponses in the visible NIR region [27]. These findings indicate that by incorporating lanthanide ions through doping and adjusting their concentrations, it becomes possible to modulate the optical absorption capability of WO_3_ inverse photonic crystals in the near-infrared region.

The photoluminescence spectra of WO_3_: x mol% Yb^3+^, 1 mol% Er^3+^ (x = 0.5, 2, 3, 4) inverse photonic crystals excited by a near-infrared 980 nm laser are presented in Figure 5b. As illustrated in Figure 5b, the upconversion luminescence intensity of WO_3_: Yb^3+^, Er^3+^ inverse photonic crystals is gradually amplified with the increasing doping concentration of Yb^3+^ ions under the excitation of a 980 nm laser. However, when the concentration of Yb^3+^ reaches 3%, the upconversion luminescence (UCL) intensity becomes saturated and diminishes upon further increases in the Yb^3+^ concentration due to the influence of the concentration quenching mechanism [28]. Therefore, the optimal doping concentration of Yb^3+^ in the WO_3_ inverse photonic crystal is determined to be 3%. As depicted, when excited by near-infrared light at 980 nm, all samples exhibit green UCL peaks at 525 nm and 552 nm emitted from the Er^3+ 2^H_11/2_/^4^S_3/2_→^4^I_15/2_ transition, and the red UCL peaks exhibited at 657 nm and 670 nm are generated by the ^4^F_9/2_→^4^I_15/2_ transitions of Er^3+^ [29,30,31]. In general, the equation *I = P^n^* can be used to investigate the UCL mechanism, where *I*, *P*, and *n* are the UCL intensity, the 980 nm laser power, and the number of photons, respectively. Figure 5c shows the red and green UCL intensities of WO_3_: 3 mol%Yb^3+^, 1 mol%Er^3+^ as a function of different laser powers. The n at 525 nm is about 2, and the n at 657 nm is about 1 because of the saturation effect due to the competition between linear decay and upconversion processes caused by the depletion of intermediate excited states [32].

Figure 5d illustrates the intricate mechanism behind the mesmerizing green and red UCL in WO_3_: Yb^3+^, Er^3+^. Under 980 nm excitation, the increase in green UCL emissions levels is mainly realized by the energy transfer process from Yb^3+^ to Er^3+^, and the electrons of the ^4^F_7/2_ energy level undergo non-radiative relaxation to reach the ^2^H_11/2_ and ^4^S_3/2_ energy levels and finally transition to the ground state ^4^I_15/2_ energy level to excite the green UCL emission. The increase in the ^4^F_9/2_ level of UCL emissions mainly comes from electrons at the ^4^I_11/2_ level undergoing non-radiative relaxation to the ^4^I_13/2_ level and then absorbing the energy of Yb^3+^ ions to transition to the ^4^F_9/2_ level. The electrons in the final excited state ^4^F_9/2_ level transition to the ground state ^4^I_15/2_ level, producing red UCL emissions. The results suggest that the UCL properties of the WO_3_ inverse photonic crystal can be modulated by adjusting the doping concentration of lanthanide ions. In conclusion, successful modulation of optical absorption capabilities in the visible and near-infrared regions, as well as the properties of upconversion luminescence of lanthanide-ion-doped inverse photonic crystals, has been achieved through lanthanide ion doping and regulation of the doping concentration, demonstrating potential applications in areas such as photocatalysis and optical storage.

### 3.3. Reversible Optical Modulation in WO_3_: Yb^3+^, Er^3+^ Inverse Photonic Crystal

**Figure 6:**
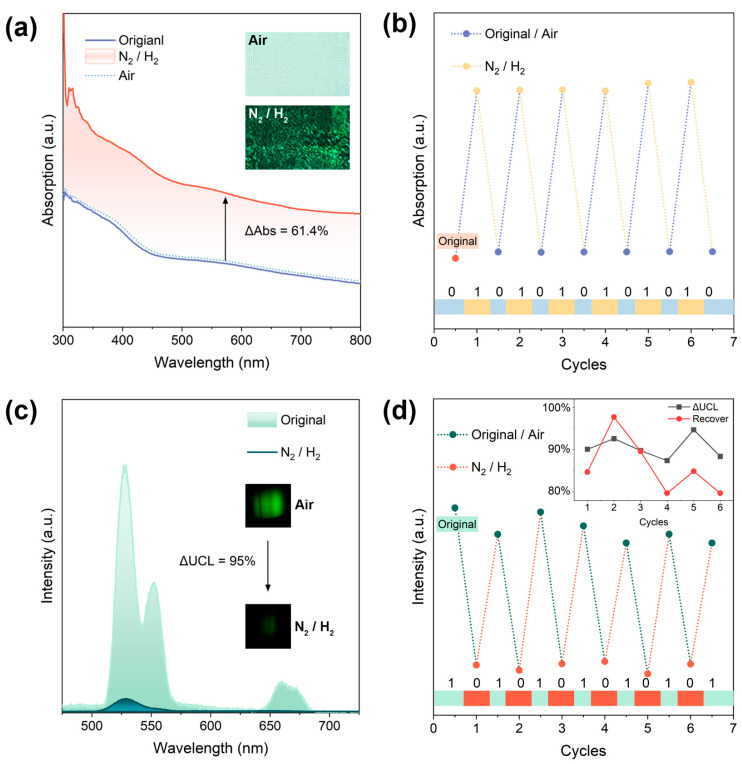
(**a**) Absorption spectra and corresponding photographs of WO_3_: Yb^3+^, Er^3+^ inverse photonic crystals in pristine state, following heat treatment in an N_2_/H_2_ atmosphere, and subsequent heat treatment in air. (**b**) Schematic diagram illustrating the absorption cycle of WO_3_: Yb^3+^, Er^3+^ inverse photonic crystals through alternating heat treatments in N_2_/H_2_ atmosphere and air. (**c**) The UCL spectra and corresponding photographs of WO_3_: Yb^3+^, Er^3+^ inverse photonic crystals before and after heat treatment in an N_2_/H_2_ atmosphere and heat treatment in ambient air. (**d**) The UCL schematic diagram illustrating the cyclic process of alternated heat treatments of WO_3_: Yb^3+^, Er^3+^ inverse photonic crystals in N_2_/H_2_ atmosphere and air.

To further explore the potential applications of WO_3_: Yb^3+^, Er^3+^ in the field of optics, the obtained WO_3_: Yb^3+^, Er^3+^ inverse photonic crystal materials were sintered at 600 °C for 3 h in an N_2_/H_2_ atmosphere in a tubular furnace. Remarkably, this treatment resulted in a transformation of the light-yellow samples into a dark green coloration. In order to assess the light absorption capacity after the heat treatment in an N_2_/H_2_ atmosphere, absorption tests were conducted on both the original and heat-treated WO_3_: Yb^3+^, Er^3+^ inverse photonic crystals, as depicted in Figure 6a. The findings unequivocally demonstrate that heat treatment under an N_2_/H_2_ atmosphere enhances the absorption capability of WO_3_: Yb^3+^, Er^3+^ inverse photonic crystals for visible and near-infrared light. This phenomenon can be attributed to a substantial increase in oxygen vacancies within the reducing environment, leading to more oxygen vacancies assisting in the accumulation of a large number of electrons and the transition from W^6+^ to W^5+^, exhibiting significant absorption promotion, especially in the NIR region, as elucidated by Lu et al.’s research [27]. The increased capacity in absorption (ΔAbs) can be characterized using Formula (1):

Δabs = (Abs_0_ − Abs_n_)/Abs_0_ × 100% (1)

where Abs_0_ and Abs_n_ represent the absorption spectral intensity of the WO_3_: Yb^3+^, Er^3+^ photonic crystal before (light yellow) and after (dark green) heat treatment, respectively. Calculations demonstrate that the ΔAbs value is 61.4%, indicating the exceptional absorption modulation capability of WO_3_: Yb^3+^, Er^3+^ in an N_2_/H_2_ atmosphere. Additionally, upon subjecting the dark green WO_3_: Yb^3+^, Er^3+^ photonic crystal to a 1 h heat treatment process in air, it is evident that the sample’s color reverts back to its initial light-yellow hue, while the absorption intensity fully regains its original strength; the corresponding bright field photographs are shown in Figure 6a. The electrons in the oxygen vacancy defects return to the valence band through the conduction band and oxidize the valence state of W to W^6+^ when exposed to atmospheric conditions, leading to the bleaching of WO_3_: Yb^3+^, Er^3+^ photonic crystal [33]. The WO_3_: Yb^3+^, Er^3+^ inverse photonic crystals also provide possibilities for electrochromism, as the principle of electrochromism is also the change in the W valence state caused by ion insertion. To further investigate the repeatability of the unique reversible color change phenomenon, the WO_3_: Yb^3+^, Er^3+^ photonic crystal was subjected to repeated cycles of heat treatment under a N_2_/H_2_ reducing atmosphere and an air atmosphere. The corresponding absorption spectral intensity for each cycle was recorded, as depicted in Figure 6b. The results suggest that through multiple iterations of this process, the amplification and attenuation of the absorption intensity in WO_3_: Yb^3+^, Er^3+^ photonic crystal can be repeatedly modulated without any degradation. This showcases exceptional reversibility and repeatability in absorption modulation, positioning WO_3_: Yb^3+^, Er^3+^ photonic crystals as highly promising materials with remarkable economic advantages, fatigue resistance, and damage resilience for practical applications in the field of optics.

Based on the realization of absorption modulation, the UCL properties of WO_3_: Yb^3+^, Er^3+^ inverse photonic crystals were evaluated by heat treatment in a reducing atmosphere, as depicted in Figure 6c. Following heat treatment in a N_2_/H_2_ reducing atmosphere for 3 h, the initial sample exhibited a significant reduction in the intense green UCL peak at 525 nm and 552 nm, along with a diminished red upconversion luminescence peak at 657 nm and 670 nm. The corresponding luminescence dark field photographs are shown in Figure 6c. According to existing research, this phenomenon can be attributed to the spectral overlap between the absorption and upconversion emission bands of WO_3_: Yb^3+^, Er^3+^ inverse photonic crystals, resulting in the quenching of upconversion luminescence [34,35]. The modulation capacity of UCL(ΔUCL) can be quantified using the following Formula (2):

ΔUCL = (UCL_0_ − UCL_n_)/UCL_0_ × 100% (2)

where UCL_0_ and UCL_n_ represent the UCL intensities of WO_3_: Yb^3+^, Er^3+^ inverse photonic crystals before (light yellow) and after (dark green) heat treatment, respectively. Calculations reveal a ΔUCL value of 95%, demonstrating the exceptional modulation capability of the UCL properties of WO_3_: Yb^3+^, Er^3+^ under different heat treatment atmospheres. Compared to powder materials, WO_3_: Yb^3+^, Er^3+^ inverse photonic crystals have a larger comparative area, which increases their contact area during heat treatment in the N_2_/H_2_ reducing atmosphere and air atmosphere, resulting in a higher optical modulation rate that is beneficial for applications such as photocatalysis, optical information storage, and electrochromism. Furthermore, as depicted in Figure 6d, this study demonstrates that the quenched UCL properties of WO_3_: Yb^3+^, Er^3+^ inverse photonic crystals can be effectively restored to their original state through heat treatment in air, enabling a reversible cycle of multiple quenching–restoration events for UCL and thus achieving remarkable luminescent modulation capabilities [36].

By integrating the reversible modulation of absorption properties and UCL properties in WO_3_: Yb^3+^, Er^3+^ inverse photonic crystals, it is feasible to achieve conditional absorption modulation in the visible and near-infrared regions, as well as adjust emission spectra under near-infrared excitation. This process exhibits reversibility and repeatability, thereby facilitating the application of WO_3_: Yb^3+^, Er^3+^ inverse photonic crystal materials in fields such as photocatalysis, optical information storage, and electrochromism.

## 4. Conclusions

In this work, the WO_3_: Yb^3+^, Er^3+^ inverse photonic crystals prepared by the template approach were investigated. The reversible and repeatable UCL and absorption modulation mechanisms were observed under alternating heat treatments in N_2_/H_2_ and air atmospheres, achieving regulation rates of absorption intensity and UCL intensity up to 61.4% and 95% due to the larger specific surface area and increased contact area for optical adjustment of the WO_3_: Yb^3+^, Er^3+^ inverse photonic crystal structure. The tunable UCL and absorption are attributed to the formation of oxygen vacancies and the transformation of W valence states under different heat treatment atmospheres. This indicates the crystal’s potential application prospects in the fields of photocatalysis, optical information storage, and electrochromism, providing guidance for the design and synthesis of new materials for future development.

## Data Availability

Data are contained within the article.
